# POSTPENSIONABLE AGE AS A DISTINCT CAREER STAGE

**DOI:** 10.1093/geroni/igad104.1149

**Published:** 2023-12-21

**Authors:** Loretta Platts, Kevin Cahill

**Affiliations:** Stockholm University, Stockholm, Sweden; Boston College, Boston, Massachusetts, United States

## Abstract

This paper presents a new, two-phase model of the late career. Drawing on life course theory, we assert the importance of age-norms for ordering the timing of people’s lives in countries with universal old-age pension systems. Three age boundaries may govern the transition to retirement: social security rules, employment rules and social norms. Upon reaching these boundaries: (1) old-age pension receipt provides a reliable income easing breadwinner pressures, (2) compulsory retirement may push older people out of the workforce, and (3) social expectations encourage participation in leisure rather than paid work. Once old-age pensions ease or eliminate bread-winning pressures, financial necessity no longer drives most of these oldest workers. Further, these working pensioners are defying social conventions calling on them to retire from paid work. Post-pensionable-age or “phase II” jobs tend to be more flexible and rewarding, and less stressful than those held by younger workers. Intriguingly, not only do these workers have quite distinctive motivations to work, they have unique ways of subduing the potential burdens of working. However, most research lumps together workers over pensionable age (in phase II) with workers still in their fifties (in phase I) who are fully exposed to labor market risks. This paper presents theoretical arguments and empirical evidence to support the idea that post-pensionable-age work is a discrete career phase economically, subjectively, and in terms of leverage with employers. It explores likely geographical and socio-economic limits to this two-phase model of the life course.

